# Synthesis and characterization of a novel CNT-FeNi_3_/DFNS/Cu(ii) magnetic nanocomposite for the photocatalytic degradation of tetracycline in wastewater†

**DOI:** 10.1039/c9ra05817f

**Published:** 2019-10-30

**Authors:** Yanhua Zhao, Jie Juan Tang, Alireza Motavalizadehkakhky, Saeid Kakooei, Seyed Mohsen Sadeghzadeh

**Affiliations:** School of Economics and Management, Langfang Normal University Langfang 065000 China; Department of Public Health and Preventive Medicine, Changzhi Medical College 046000 China; School of Humanities, Tianjin Agricultural University Tianjin 300384 China daishuxue2000@126.com; Department of Chemistry, Faculty of Sciences, Neyshabur Branch, Islamic Azad University Neyshabur Iran amotavalizadeh@yahoo.com; Centre for Corrosion Research, Department of Mechanical Engineering, Faculty of Engineering, Universiti Teknologi PETRONAS 32610 Seri Iskandar Perak Darul Ridzuan Malaysia; New Materials Technology and Processing Research Center, Department of Chemistry, Neyshabur Branch, Islamic Azad University Neyshabur Iran; Young Researchers and Elite Club, Neyshabur Branch, Islamic Azad University Neyshabur Iran

## Abstract

Herein, Cu(ii) complexes were anchored within the nanospaces of a magnetic fibrous silicate with a high surface area and easily accessible active sites *via* a facile approach, leading to the successful synthesis of a novel potent nanocatalyst (FeNi_3_/DFNS/Cu). Furthermore, FeNi_3_/DFNS/Cu was supported on carbon nanotubes (CNTs) *via* an usual nozzle electrospinning method (CNT-FeNi_3_/DFNS/Cu). In addition, its performance as a photocatalyst for the degradation of tetracycline was tested in a batch reactor. Tetracycline is an antibiotic that is commonly utilized in veterinary medicine and in the treatment of human infections, but is hazardous to aquatic environments. However, the usual processes for the removal of tetracycline are not efficient. The eco-friendly attributes of this catalytic system include high catalytic activity and ease of recovery from the reaction mixture using an external magnet, and it can be reused several times without significant loss in its performance. Also, protocols such as hot filtration, and mercury poisoning provided complete insight into the nature of this heterogeneous catalyst.

## Abstract 2

Herein, Cu(ii) complexes were anchored within the nanospaces of a magnetic fibrous silicate with a high surface area and easily accessible active sites *via* a facile approach, leading to the successful synthesis of a novel potent nanocatalyst (FeNi_3_/DFNS/Cu).

## Graphical abstract

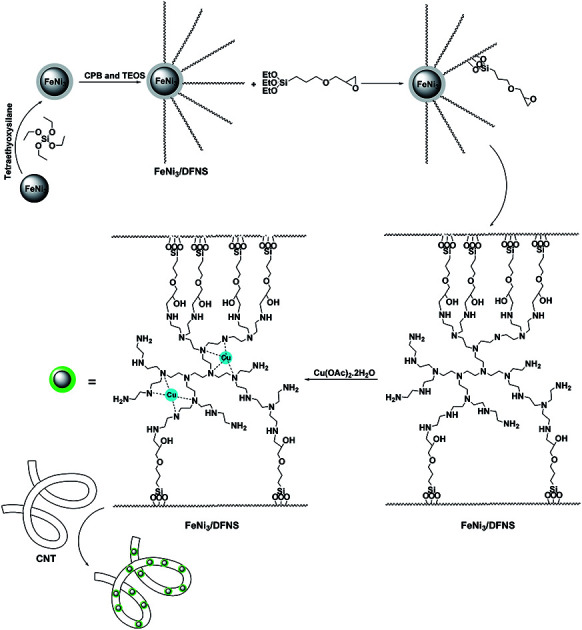

## Introduction

Tetracycline is used as an antibiotic for the treatment of human and animal infections,^1–3^ and thus wastewater commonly contains tetracycline due to its disposal. Antibiotics can cause allergies in humans. TC has superior structural strength and is resistant to biological degradation.^4–6^ Thus, antibiotics such as TC pose a great threat to the environment due to their long-term presence.^7,8^ Hence, the identification and remediation of these environmental pollutants should be prioritized. The traditional methods for the detection of TC involve chromatographic,^11^ spectroscopic,^10^ and microbial^9^ measurements, which are inefficient since they are time consuming, inaccurate and require prototype pre-tests that ultimately increase the overall cost.^12–15^ The typical techniques for the removal of tetracycline include biological degradation, ion exchange, degradation by photocatalysis, and membrane filtration and adsorption.^16^ Additionally, advanced oxidation processes, photo-electrocatalysis, reduction by zero iron nanoparticles, use of pulse radionuclides and carbon nanotubes, ozonation with ultrasonic and light treatment, and oxidation using Fe–Mn dual oxides have been reported.^17–21^

Among them, increasing interest has been focused on the application of advanced oxidation processes for the treatment of antibiotics in water, such as UV/H_2_O_2_ process,^22,23^ electrochemical methods,^24^ electro-Fenton processes,^25^ photocatalysis^26^ and photo-Fenton-like oxidation.^27^ In particular, ozonation is capable of oxidizing organic compounds to simpler and more easily biodegradable compounds,^28–30^ such as procaine penicillin G,^30^ amoxicillin,^31,32^ ceftriaxone sodium,^33^ macrolide,^34^ sulfonamide,^34,35^ penicillin^36^ and tetracycline.^37–39^ However, almost all the research has been performed in conventional bubble column reactors.

In society, due to the demand for economic and ecologically viable procedures, the development of supported catalysts is the focus of several studies. Carbonaceous materials are commonly employed as suitable candidates in the synthesis of supported catalysts.^40–43^ Nevertheless, the commonly utilized carbonaceous supports suffer from various disadvantages such as the presence of different impurities, which may poison the catalyst, and a microporous structure, restricting mass-transfer. Moreover, their lack of spectroscopic properties can hinder the basic understanding of the resulting materials. In order to overcome these problems, nano-carbons have been demonstrated as a promising option in the preparation of supported catalysts.^44,45^ Basically, because of their prominent characteristics including high surface area and high chemical and thermal stability, carbon nanotubes (CNTs) have been widely investigated as catalyst supports.^46,47^

Recently, the use of surfactants in soft templating has led to the production of mesoporous silica with a dendritic silica fiber morphology (DFNS). Silica possessing this morphology has been investigated for catalysis and adsorption processes as a support material. The outward radial widening of these silicas cause them to possess a higher surface area for reactants to access the functional materials more efficiently. In addition, DFNS has intrinsic mesoporous properties, thermally stability and high activity. The synthesis of DFNS requires a microemulsion system containing a surfactant, oil and water. Furthermore, the particle size and morphology of DFNS can be easily manipulated by introducing a co-surfactant and various cosolvents.^48–54^

The FeNi_3_ alloy has magnetic properties and is widely utilized due to its high permeability, low energy loss, high temperature and high saturation magnetism. Nevertheless, it is a poor nanoadsorbent, and thus researchers commonly utilize functionalized metal oxides and polymers as well as antioxidants. The coating of SiO_2_ with FeNi_3_ nanoparticles may impressively enhance their electrical properties at high-frequency. Currently, distinct approaches are utilized for providing nanocomposites of magnetic alloys and their nanoparticles. Nevertheless, in most cases, their preparation requires strict conditions such as vacuum, inert atmosphere and sometimes hydrogen, high pressure and temperature. Also, the risks of Fe nanoparticles need to be further studied.^52–55^

Cu(ii) metal complexes have attracted significant attention recently because of their simple electronic configuration, structural diversity, distorted coordination geometry, and applications in catalysis, biochemistry, medicine and sensing. A plethora of multinuclear metal complexes has been reported. The choice of the bridging ligands, metal ions and terminal ligands is considered essential in the rational design of multinuclear metal complexes with particular physicochemical characteristics. Bridging ligands to nitrogen and sometimes active oxygen transfer atoms such as oxo/alkoxo,^56^ oxalate,^57^ carboxylate,^58^ sulfato^59^, hydroxo,^60^ phenoxo,^61^ azido^62^ and dicyanamide^63^ have been extensively utilized to produce multinuclear homo- and hetero-metallic Cu(ii) complexes. Nevertheless, the oxalate dianion has been considered a noteworthy bridging ligand for producing diverse types of homo- and hetero-metallic Cu(ii) complexes with promising physical and chemical characteristics such as magnetic, catalytic, and optical properties.

In the present study, it is hypothesized that the open and fibrous morphology of DFNS can facilitate the loading of a high content of Cu(ii) complexes with minimal loss in surface area. Therefore, enhancing the availability of Cu(ii) sites can enhance the light harvesting ability of the complex because of improved scattering and also reflection of the incident light because of the fibrous internal structure in the catalyst. It can also lead to efficient interaction among the produced charges and the reactants because of the proximity of the excitons and adsorbed molecules at the surface of the Cu(ii) complexes located on the DFNS fibers. Additionally, due to the adverse influence of antibiotics in the environment, the nanocomposite was utilized for the treatment of water based on its high performance and the development of oxidation approaches for the removal of organic containments. Thus, the purpose of this work was to synthesize the DFNS/Cu(ii) nanocomposite for the photocatalytic adsorption of tetracycline. In addition, we studied the influence of various factors on the activity of the nanocomposite including pH, dose, contact time and tetracycline concentration. Also, carbon nanotubes (CNTs) were selected as a supporter for the nanoscale composition of FeNi_3_/DFNS/Cu nanoparticles. The benefits of utilizing CNTs as a supporter for spreading the metal catalyst particles are illustrated to be because of the enrichment in the level functional groups and defects. Besides their availability in the active stage and proper chemical consistency in harsh media, the single electronic structure of CNTs displays a great charge transfer that kinetically reduces the diffusion resistance.

## Experimental

### Materials and methods

High purity chemicals were procured from Fluka and Merck. Electrothermal 9100 apparatus were utilized for the determination of uncorrected melting points in open capillaries. VERTEC 70 spectrometer (Bruker) in transmission mode were used for the determination of FTIR spectra. Samples were pulverized and pelletized with spectroscopic grade KBr. Determination of size and structure of nanoparticles were done *via* transmission electron microscope (TEM) (Phillips CM10) operated at 100 kV. The crystallographic structures of nano particles were determined using powder X-ray diffraction (Bruker D8 Advance model) with Cu Kα radiation. Thermal gravimetry analysis (TGA) (NETZSCH STA449F3) was utilized under nitrogen atmosphere with a heating rate of 10 °C min^−1^. Thin later chromatography (TLC) done on silica gel polygram SILG/UV 254 plates were used for the determination of product purity and monitoring of reaction.

### General procedure for the preparation of FeNi_3_ MNPs

The procedure for the preparation of the FeNi_3_ MNPs involved dissolving 0.01 mol FeCl_2_·4H_2_O and 0.03 mol NiCl_2_·6H_2_O in 300 mL of distilled water and the subsequent addition of 1.0 g of polyethylene glycol (PEG, MW 6000). To achieve the pH range of 12 ≤ pH ≤ 13, sodium hydroxide (NaOH) was added to the solution. Next, hydrazine hydrate (N_2_H_4_·H_2_O) at 80% concentration was added to the suspension in varying quantities. The suspension was left to react continuously for 24 h at room temperature. The pH of the suspension was maintained at 12 ≤ pH ≤ 13 with periodical dosing of NaOH. Subsequently, the solid phase of the resultant mixture was filtered to obtain the black colored FeNi_3_ MNPs and rinsed with ionized water multiple times.

### General procedure for the preparation of FeNi_3_/SiO_2_ MNPs

An aqueous solution containing 80 mL of ethanol, 20 mL of ionized water and 2.0 mL of 28 wt% concentrated ammonia aqueous solution (NH_3_·H_2_O) was prepared. 0.02 mol of FeNi_3_ MNPs was dispersed in the mixture and 0.20 g of tetraethyl orthosilicate (TEOS) was added subsequently. The mixture was vigorously stirred for 24 h. The solid phase of the resultant mixture was filtered and washed several times before being dried in atmospheric conditions at 60 °C.

### General procedure for the preparation of FeNi_3_/DFNS MNPs

Solution A was prepared by adding 30 mL aqueous solution containing 0.3 g urea and dispersing 0.25 g of FeNi_3_/SiO_2_. The mixture was placed in an ultrasonic bath for 1 h. Solution B was prepared by adding 0.5 g cetylpyridinium bromide (CPB) to 0.75 mL of *n*-pentanol and 30 mL of cyclohexane. Solution A and B were mixed and stirred at room temperature and 1.25 g TEOS was added dropwise. The mixture was continuously stirred for 1 h at room temperature and placed in an oven at 120 °C for 5 h to initiate the reaction. After the reaction was complete, the mixture was cooled to room temperature before applying a strong magnetic suction for the isolation of the FeNi_3_/DFNS core–shell microspheres. The solid phase was then washed several times with water and acetone. The washed solid was dried in a drying oven at 40 °C overnight and calcined at 550 °C for 5 h in atmospheric condition.

### General procedure for the preparation of FeNi_3_/DFNS/GMSI MNPs

A mixture was prepared by mixing 200 mg of FeNi_3_/DFNS in 20 mL of THF followed by the addition of 20 mmol of NaH *via* ultrasonication. Subsequently, 22 mmol of (3-glycidyloxypropyl)trimethoxysilane was added to the mixture at room temperature and stirred for 16 h at 50 °C. The resultant mixture was filtered and washed with ethanol and deionized water. The filtered solid was then dried under vacuum at 50 °C for 3 h.

### General procedure for the preparation of FeNi_3_/DFNS/PEI MNPs

Some FeNi_3_/DFNS, about 100 mg, activated by glycidyloxypropyl was released in 10 mL of acetate buffer (0.1 M and pH 4.5) comprised of 50 mg of poly(ethylene imine) (PEI). The suspension was shaken at 120 rpm for a few hours to produce nanoparticles containing PEI. Using acetate buffer, the resultant nanoparticles were washed several times and stored at a temperature of 4 °C.

### General procedure for the preparation of FeNi_3_/DFNS/Cu MNPs

A mixture of FeNi_3_/DFNS/PEI (50 mg) and Cu(OAc)_2_·2H_2_O (4 mmol) in methanol (20 mL) was stirred at room temperature under an argon atmosphere. After a reaction time of 1 h, the complex was formed.

### Common method for the synthesis of CNT-FeNi_3_/DFNS/Cu nanoparticles

0.40 g of CNT was dispersed in 50 mL water ultrasonically, and then 80 mg of FeNi_3_/DFNS/Cu MNPs was added. After stirring and ultrasonic dispersal for around 120 min, CNT-FeNi_3_/DFNS/Cu was obtained using a magnet and then dried under vacuum.

### Adsorption and photocatalytic removal of TC experiments

A 1000 mg L^−1^ stock solution was prepared by dissolving TC hydrochloride salt in deionized water. The initial pH (pH_initial_) of the sodium chloride solution was adjusted between 2 and 12 *via* the addition of 0.1 M hydrochloric acid (HCl) or 0.1 M sodium hydroxide (NaOH) solutions. 5 mg of CNT-FeNi_3_/DFNS/Cu photocatalyst was added to the solution and shaken at 300 rpm for 180 min. For the determination of pH_zpc_, pH_final_ was measured and plotted against pH_initial_. The point where the curve crossed the line pH_initial_ = pH_final_, gave pH_zpc_. Adsorption experiments were carried out on 200 mL sample in batch at temperatures ranging from 5–50 °C. The photocatalytic experiment was also carried out in batch on 400 mL sample at room temperature (24 ± 2 °C) in a 500 mL UV reactor.

## Results and discussion

**Scheme 1:**
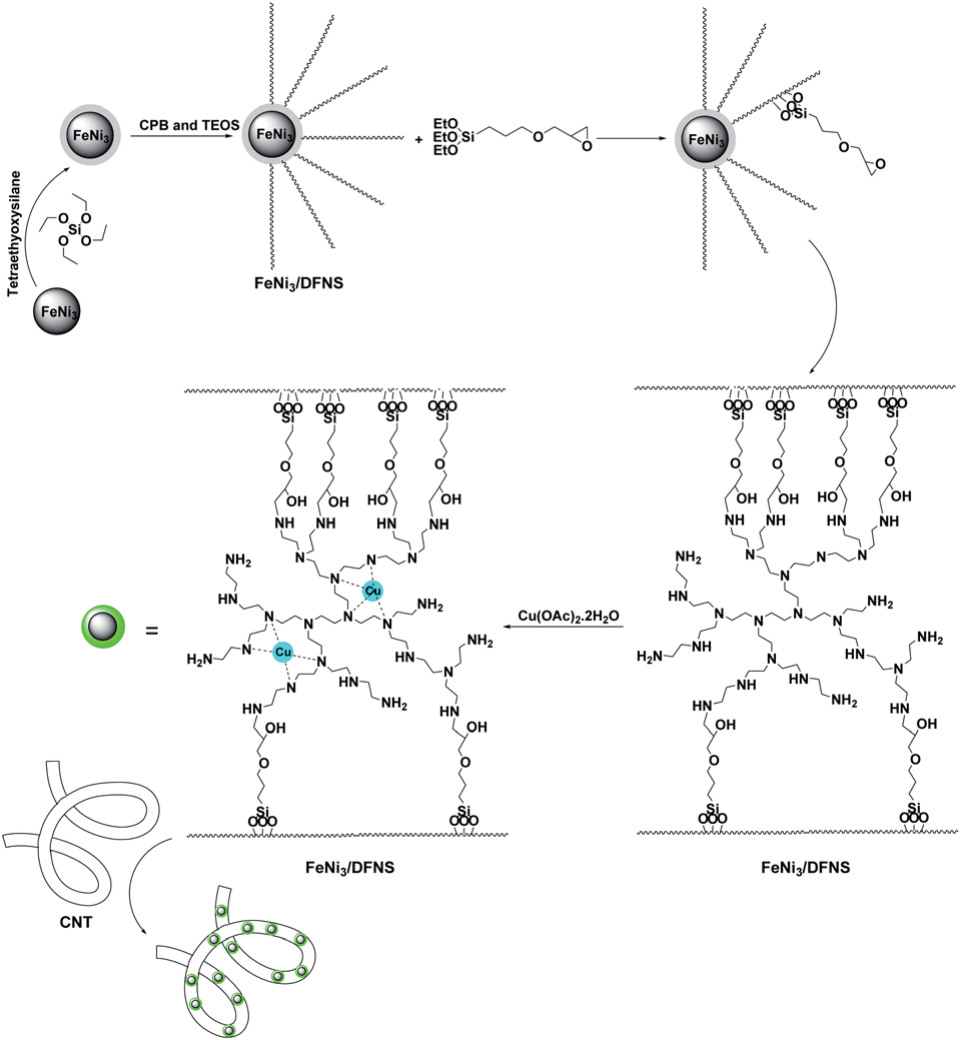
Preparation of CNT-FeNi_3_/DFNS/Cu nanocatalyst.

In the present work, the magnetic DFNS solution was produced using reported methods and then modified using (3-glycidyloxypropyl)trimethoxysilane, followed by reductive amination to obtain the corresponding co-immobilized copper(ii) complexes. For the preparation of the CNT-FeNi_3_/DFNS/Cu nanocatalyst, CNT were functionalized with mercaptopropyl groups so that the FeNi_3_/DFNS/Cu MNPs could be easily anchored on them (Scheme 1).

The MNP complexes of FeNi_3_/DFNS and FeNi_3_/DFNS/Cu(ii) with a distinct morphology and physical structure were investigated *via* FESEM and TEM, as shown in Fig. S1.† As can be observed in Fig. S1a and c,† FeNi_3_/DFNS has silica fibres, a nonporous silica layer, and a core of FeNi_3_ particles. In addition, the samples possess spheres of identical size with diameters of around ∼200 nm and a corrugated radial structure. Analysis of the TEM and FESEM images showed that the FeNi_3_/DFNS/Cu(ii) complex contained dendritic fibers with a thickness of around 5–8 nm arranged in three dimensions, producing walls that may lead to easy availability for the appropriate molecules. As can be observed in Fig. S1b and d,† the FESEM and TEM images of the FeNi_3_/DFNS/Cu(ii) complex MNPs indicate after varying the MNPs their morphology did not change.

The FTIR spectrum demonstrates the presence of surface phosphate, hydroxyl, and silanol groups from the organic groups in (a) FeNi_3_/DFNS, as well as (b) the amino-functionalized nanoparticles of FeNi_3_/DFNS in Fig. S2.† In the case of FeNi_3_/DFNS, the wide absorption bands at 1089 cm^−1^ and 3654 cm^−1^ are ascribed to the Si–O–Si unsymmetrical stretching and OH, respectively. The two peaks located at 799 cm^−1^ and 467 cm^−1^ are ascribed to the Si–O–Si symmetrical stretching and bending, respectively (can be seen in Fig. S2a†). These results clearly indicate the grafting of GMSI on the surface of FeNi_3_/DFNS. The FeNi_3_/DFNS/PEI composite shows bands at around 1091, 793 and 462 cm^−1^. The wide absorption band at around 3000–3550 cm^−1^ is related to the –OH and –NH stretching vibrations. The peak at around 2930 cm^−1^ appeared due to the stretching of the C–H aliphatic group (can be observed in Fig. S2b†).

The analysis of nitrogen physisorption indicated that the specific BET surface area of the FeNi_3_/DFNS and FeNi_3_/DFNS/Cu(ii) complexes was 679, and 341 m^2^ g^−1^, respectively. The decrease in the surface area of the FeNi_3_/DFNS/Cu(ii) complex in comparison with that of FeNi_3_/DFNS can be due to the Cu(ii) complexes. The nitrogen adsorption–desorption isotherms of the FeNi_3_/DFNS supported catalysts can be seen in Fig. S3.† FeNi_3_/DFNS exhibited a type IV isotherm, with an H1-type hysteresis loop, indicating the existence of mesopores. The related pore size section predicted by the desorption branch of the nitrogen isotherm by the BJH procedure displayed a narrow pore distribution with the maximum at 9 nm (Table 1). The large mesopore size of FeNi_3_/DFNS with high capacity may load Cu(ii) complexes, which have a comparatively large molecular size.

**Table 1:** Structural parameters of FeNi_3_/DFNS and FeNi_3_/DFNS/Cu(ii) complex MNPs

Catalyst	*S*_BET_ (m^2^ g^−1^)	*V*_a_ (cm^3^ g^−1^)	*D*_BJH_ (nm)
FeNi_3_/DFNS	679	3.3	9
FeNi_3_/DFNS/Cu(ii)	341	1.9	4

The level roughness of the FeNi_3_/DFNS/Cu(ii) MNPs in terms of their fibrous state was also investigated *via* atomic force microscopy (AFM), and the topographic images are illustrated in Fig. S4.† As can be observed in Fig. S4,† the highest regions, indicated by the brighter yellowish white color, were enhanced by decreasing T/W, suggesting an increase in the roughness of the catalyst surface.

Fig. S5† shows the cyclic voltammograms of the Cu(ii) complex for around 5 mmol with 100 mmol tetramethylammonium nitrate (TMAN) in DMSO at 25 °C, in the potential range of −1.4 to +0.1 V with a scan range of around 100 mV s^−1^. The complex exhibited a signal that is quasi-reversible in the negative zone, specific of the couple of Cu(ii) → Cu(i) in *E*_pc_ = −0.880 V. The anodic peak indicated in *E*_pa_ = −0.529 V is for oxidation reaction (Cu(i) → Cu(ii)). The reversible conduct shows that complexes of Cu(ii) and Cu(i) are Schiff bases and are stable in DMSO under the surface of the electrode.^64^

In addition, in the case of the nanofibrous composite of CNT-FeNi_3_/DFNS/Cu(ii), the TEM images of the pure nanofibers with various diameters indicated their identical structures as observed in Fig. S6a.† Subsequently, they nucleate and develop *via* the electrospinning method, and FeNi_3_/DFNS/Cu(ii) changed to nanofiber CNT due to the internal radial direction of the electrostatic area and the fast evaporation of the solvents. Actually, Fig. S6a† indicates the high-resolution TEM picture of the CNT-FeNi_3_/DFNS/Cu(ii) nanofiber and the short spaces between the separated straight chains. The chains of the CNT are extended and also self-oriented in the direction along the fiber axis and the straight molecular sections were 800–900 nm long in these chains. Moreover, as seen in Fig. S6b,† the SEM image of CNT-FeNi_3_/DFNS/Cu(ii) proves that the typical white and gray dots located onto the direct chain of CNT are FeNi_3_/DFNS/Cu(ii). The compression of FeNi_3_/DFNS/Cu(ii) is demonstrated and its mean size was computed to be around 200 nm.

The X-ray diffraction patterns of the MNP complexes of FeNi_3_/DFNS/Cu(ii), CNT-FeNi_3_/DFNS/Cu(ii) are illustrated in Fig. S7.† As seen, all the considered samples have the generic diffraction peaks at around (111), (200), and (220), which are consistent with the information for the modulus sample of FeNi_3_, as previously stated in the JCPDS Card (No. 19-0629), as can be seen in Fig. S7a.† As can be seen in Fig. S7b,† the peak of iron oxide and the XRD pattern of the core shell nanoparticles of FeNi_3_/DFNS/Cu(ii) exhibited a wide featureless peak in the low diffraction angle, which is ascribed to the silica in its amorphous state.

XPS was utilized to study the chemical composition of the MNPs of CNT-FeNi_3_/DFNS/Cu(ii). The XPS pattern for the as collected catalyst is shown in Fig. S8,† which show peaks related to O, Cu, Si, N, I, Fe, Ni, and C, and the presence of N 1s was confirmed, which is due to the functionalization of DFNS using PEI. In addition, the XPS spectrum of Cu 2p shows a doublet indicating metallic Cu. The origins in the catalyst were detected using EDX analysis (refer to Fig. S9†). As can be seen in Fig. 9, the EDX pattern demonstrates all the elements existing in the MNPs of CNT-FeNi_3_/DFNS/Cu including, nickel carbon, silicon, nitrogen, oxygen, copper, and iron.

Thermogravimetric analysis (TGA) is known as a strong method for predicting the functionalization percentage of CNTs sites to verify the composition of the supported catalyst. As can be seen in Fig. S10,† the raw CNTs exhibited no significant weight loss. In contrast, the functionalized carbon nanotubes exhibited a weight loss of around 8% at the temperature of 150 °C due to the elimination of adsorbed water. The weight loss of around 24% in the temperature range of 150 °C to 600 °C is because of the removal of the amino groups from the CNT sites. The results of our experiments indicate that around 24% of organometallic groups were added on the CNT sites.

A vibrating sample magnetometer (VSM) was utilized to investigate the magnetic characteristics of the nanoparticles with the magnetization diagrams of the nanocomposite obtained at a temperature of 300 K. As seen in Fig. S11,† no magnetism was discovered; thus, the nanocomposites have paramagnetic properties. The saturation magnetization of 45.2 and 19.7 emu g^−1^ was calculated for FeNi_3_/DFNS/Cu(ii) and CNT-FeNi_3_/DFNS/Cu(ii) MNPs, respectively. An external magnetic zone together with the ability for quick scattering upon removal of the magnetic zone is a property of paramagnetic nanocomposites with high magnetization values. Therefore, the resultant nanocomposite presented suitable magnetic responsivity for its potential usage in targeting and for separation.

For UV irradiation, a lamp with nominal capacity of 18 W with a wavelength of 254 nm and radiation intensity of 2500 μW cm^−2^ was used. It was placed in the center of the reactor. To maintain the sample temperature inside the reactor within the temperature range of 24 °C ± 2 °C, a cooling-water wall was used around the reactor. The samples were withdrawn at certain intervals, and the measurement of the concentration of the remaining tetracycline was performed using a spectrophotometer (T80^+^ UV/Visible, PG, UK) at a wavelength of 358 nm (Fig. S12†).

**Fig. 1:**
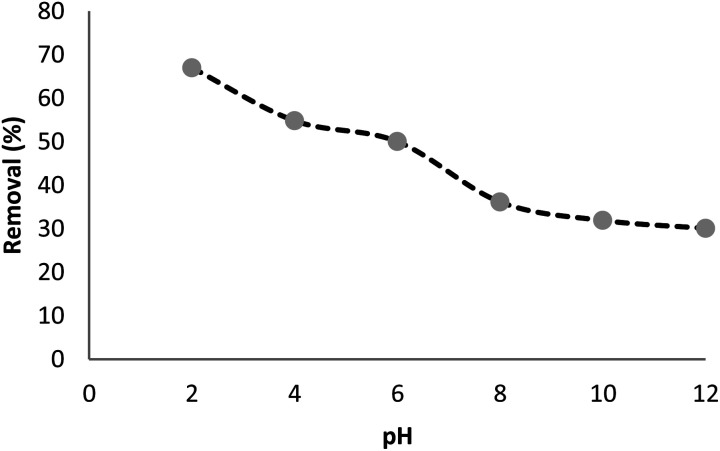
Tetracycline adsorption by CNT-FeNi_3_/DFNS/Cu(ii) nanocomposite with different pH.

**Scheme 2:**
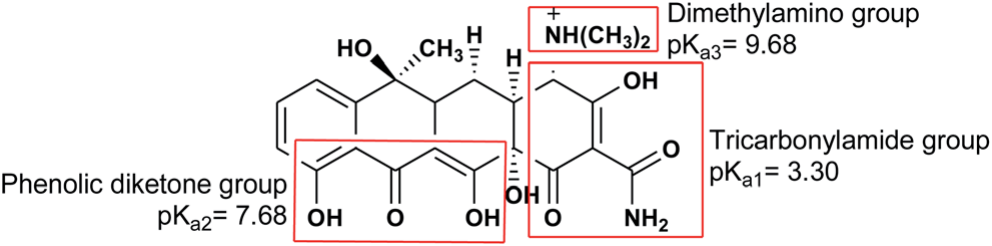
Structure of TC with its three acidic dissociation constants (p*K*_a_).

**Fig. 2:**
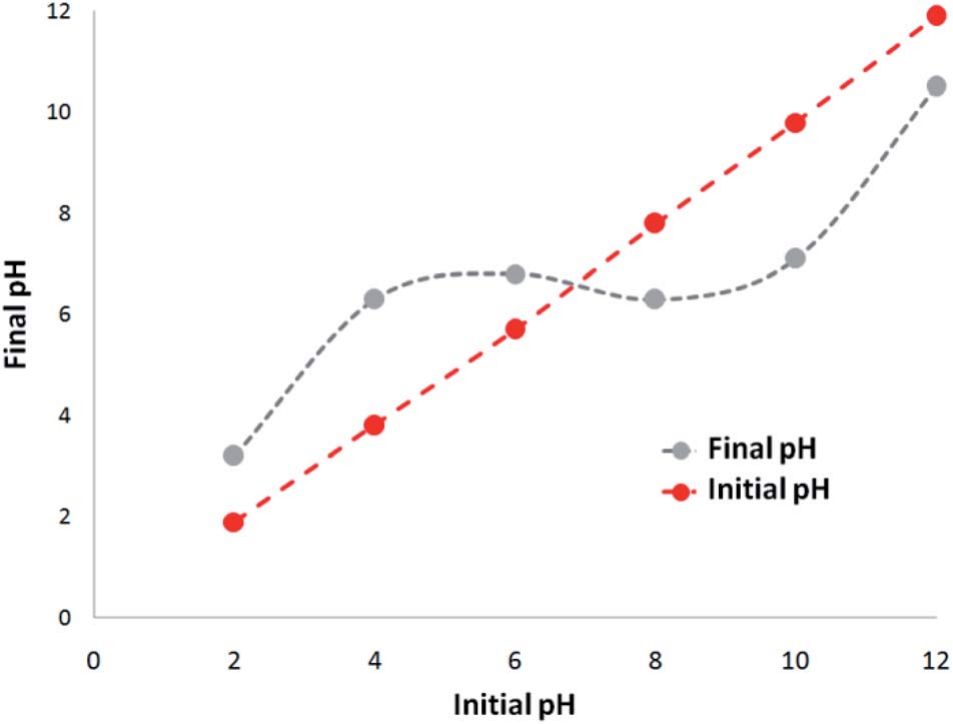
Zeta potential of CNT-FeNi_3_/DFNS/Cu(ii) at different pH values.

The absorption of TC by the CNT-FeNi_3_/DFNS/Cu(ii) MNPs was investigated by varying several parameters such as contact time, amount of nanocatalyst, concentration of tetracycline, pH, and temperature. The absorption of tetracycline by the nanocomposite at different pH was determined with 100 mL sample and an absorbent concentration of 10 mg L^−1^ at room temperature, as shown in Fig. 1. As can be seen, the absorption of TC at 1 h at pH = 2, 4, 6, 8, 10, and 12 is 66.98%, 54.81%, 50.02%, 36.14%, 31.89%, and 30.08%, respectively. This can be attributed to the electrical charge of tetracycline and the superficial charge of the absorbent at various pH. In an acidic environment, H^+^ ions are bombarded on the absorbing surface. Therefore, negatively charged ions are easily adsorbed on this type of structure. Phenolic, ketone, carboxylic, amine and alkylic groups are present in tetracycline in alkaline, neutral and acidic environments. Based on Scheme 2, TC has three types of acid decomposition (p*K*_a_). In an acidic environment, proton bombarding of dimethylamine in TC causes it to be positively charged and it has negative/positive values at pH values of 3.8 to 7.8 due to its neutralization. Thus, when TC is in a neutral environment, a quantity of protons in the phenotype diketone is lost. Eventually, TC has a negative charge in alkaline conditions. pH_ZPC_ is the state where initial pH = final pH. Based on the results, the efficiency in TC removal using the CNT-FeNi_3_/DFNS/Cu(ii) absorbent is affected by a change in pH. At pH less than 6, more of the functional groups in TC have a negative charge, whereas the CNT-FeNi_3_/DFNS/Cu(ii) surface is positively charged. Thus, electrostatic adsorption between the positive surface of the adsorbent and negative TC functional groups will increase the absorption efficiency. Consequently, as pH increases to 10, the presence of OH^−^ resulted in a decrease in the adsorption. In addition to the negatively charged TC functional groups, the amount of adsorption by the catalyst is reduced (Fig. 2).

**Fig. 3:**
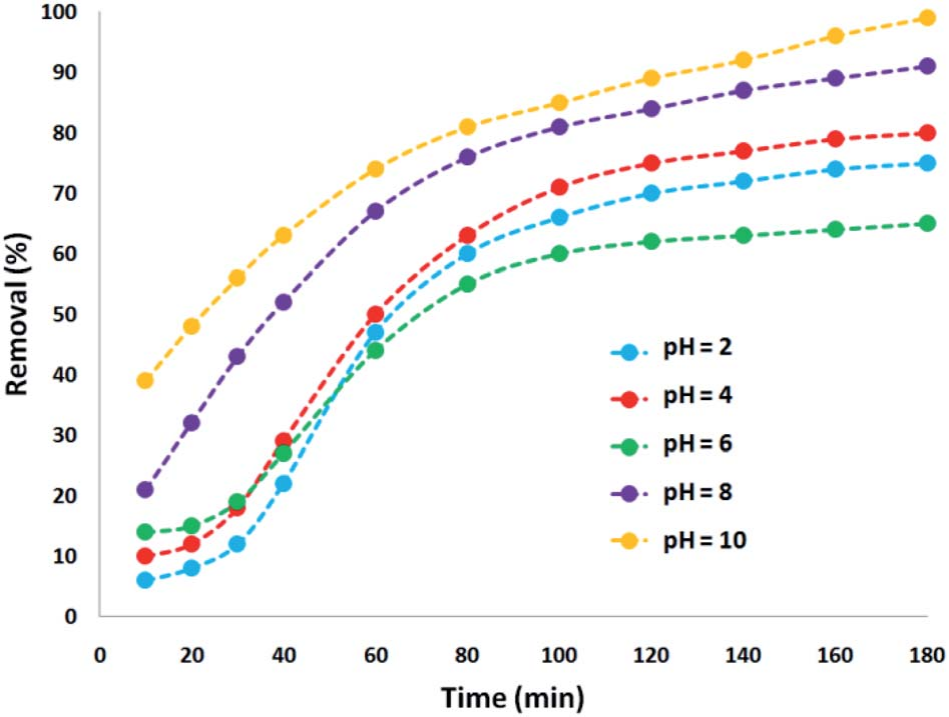
Tetracycline degradation *via* nano-photocatalysis with the CNT-FeNi_3_/DFNS/Cu(ii) MNPs at different pH.

According to previous studies, the pH of the reaction environment plays an essential role in the removal and destruction of TC. In oxidation reactions, absorption capacity, distribution of electric charge on the catalyst surface, valence band oxidation potential and degradation speed of the pollutant are greatly impacted by pH. In another study, pH was found to notably affect the destruction of tetracycline. It has been reported that at pH = 2 and 10, the minimum and maximum percentage of destruction were obtained, respectively. According to the results, the photocatalytic destruction of tetracycline best occurred in an alkaline environment (Fig. 3).

The results of previous studies showed that an increase in the pH of the environment increases the quantum yield of tetracycline. Tetracycline with several functional groups is an amphoteric compound with multiple ionizing sites. As aforementioned, in varying pH environments, tetracycline with different main groups has several p*K*_a_. Based on Scheme 2, three protonated main groups are found in the tetracycline structure, which include dimethyl amino, phenolic diketone, and tricarbonyl methane groups. Thus, the destruction rate and quantum efficiency of tetracycline at varying pH can be caused by the dominance of one or several forms of tetracycline in varying environmental pH. Previous research also reported similar results,^65^ showing the least tetracycline degradation in a photocatalytic reaction in the presence of H_4_TC^+^.

**Fig. 4:**
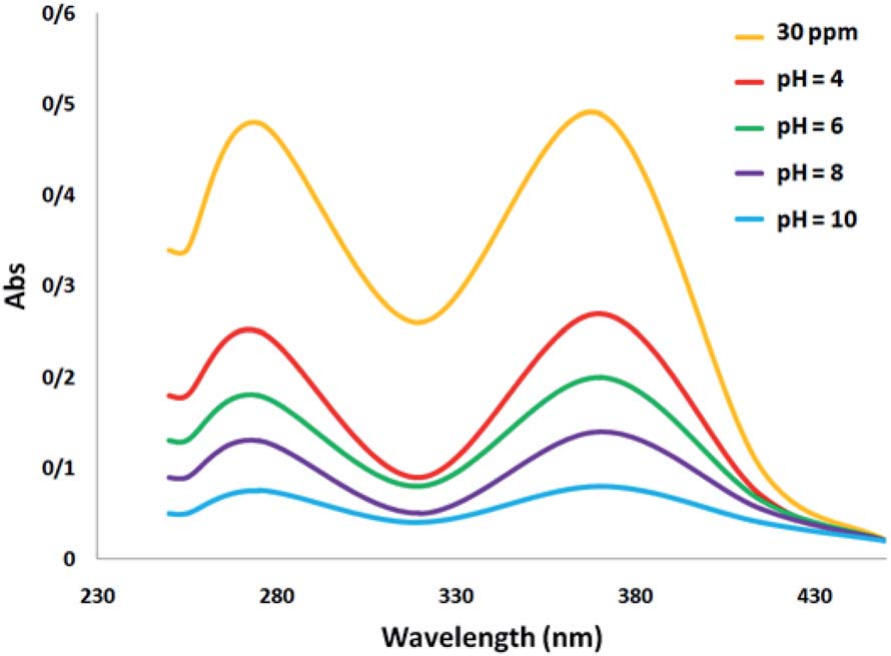
Comparison of the UV-Visible spectra of TC at different pH.

Fig. 4 shows the absorption spectrum associated with the photocatalytic degradation of tetracycline with a change in pH. It can be observed that the biggest peak corresponds to the initial dose of tetracycline. Changing the pH has the effect of changing the height of the peak, which is an indication of the removal of the antibiotic. Specifically, an increase in the percentage of tetracycline removal is confirmed by the decrease in peak height at alkaline pH.

**Fig. 5:**
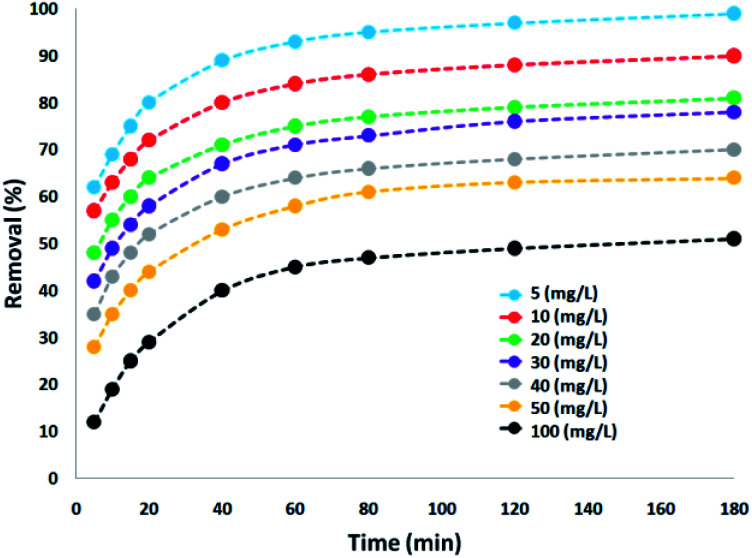
Effect of nanocomposite dosage in the presence of UV light on tetracycline degradation.

The effect on the amount of CNT-FeNi_3_/DFNS/Cu(ii) MNPs on the destruction of tetracycline was tested within the range of 5–100 mg L^−1^ under ultraviolet light. The experiments were performed on 30 mg L^−1^ tetracycline at pH = 10. The degradation yield of tetracycline decreased under ultraviolet light with an increase in the concentration of the CNT-FeNi_3_/DFNS/Cu(ii) MNPs, where after 180 min, at 100 and 5 mg L^−1^, the degradation yield decreased to 51% and 99%, respectively. This is consistent with that observed in several reports,^66^ which is due to the increment in opacity as the dosage of CNT-FeNi_3_/DFNS/Cu(ii) MNPs increased. It causes a disorderly transmission of light in the solution,^67^ leading to a reduction in the efficiency for the removal of tetracycline. In addition, the decrease in removal efficiency with an increase in the amount of catalyst can be caused by the agglomeration of the nanocatalyst at high concentration. This reduces the active sites for photons to be absorbed on its surface. The degradation performance exhibited a high rate in the initial 80 min. The percentage of destruction increased significantly, but the tetracycline degradation percentage with an increase in photocatalytic time did not result in significant changes, and thus, the speed of antibiotic removal from the wastewater did not have a significant increase. According to the results, the optimal amount and time for the reaction were 5 mg and 180 min, respectively, which is very affordable. Because of different anions, such as chlorine, sulfate, phosphate, and bromine, low light penetration occurred in the reaction, which reduced the reaction progress (Fig. 5).

**Fig. 6:**
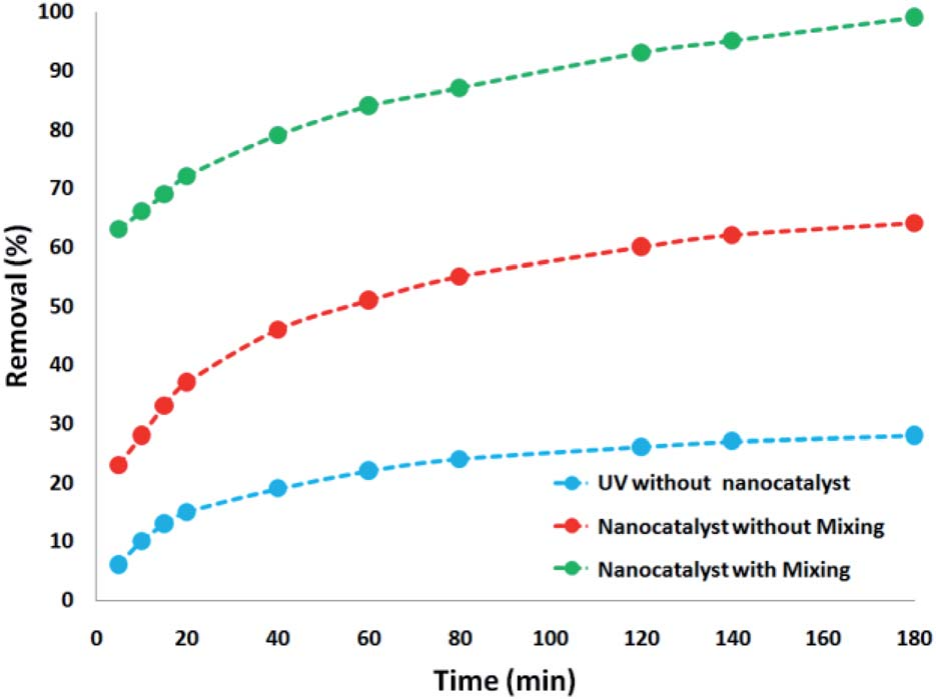
Comparison of tetracycline degradation in various processes with optimal conditions.

Thus, to decrease the opacity and determine the effect of the CNT-FeNi_3_/DFNS/Cu(ii) MNP dose in the elimination of contamination under ultraviolet light, the blending factor was removed by turning off the magnetic stirrer as the nanocatalyst was added to the UV pilot reactor. It was determined that despite the increment experienced in the reduction percentage with an increase in the CNT-FeNi_3_/DFNS/Cu(ii) MNP amount, it was negligible in comparison with the results obtained from the UV pilot reactor with stirring. This is because the maximum percentage of pollutant removal using the nanocatalyst with stirring was 99%. This difference in efficiency is quite evident compared to during the absence of stirring (Fig. 6).

**Fig. 7:**
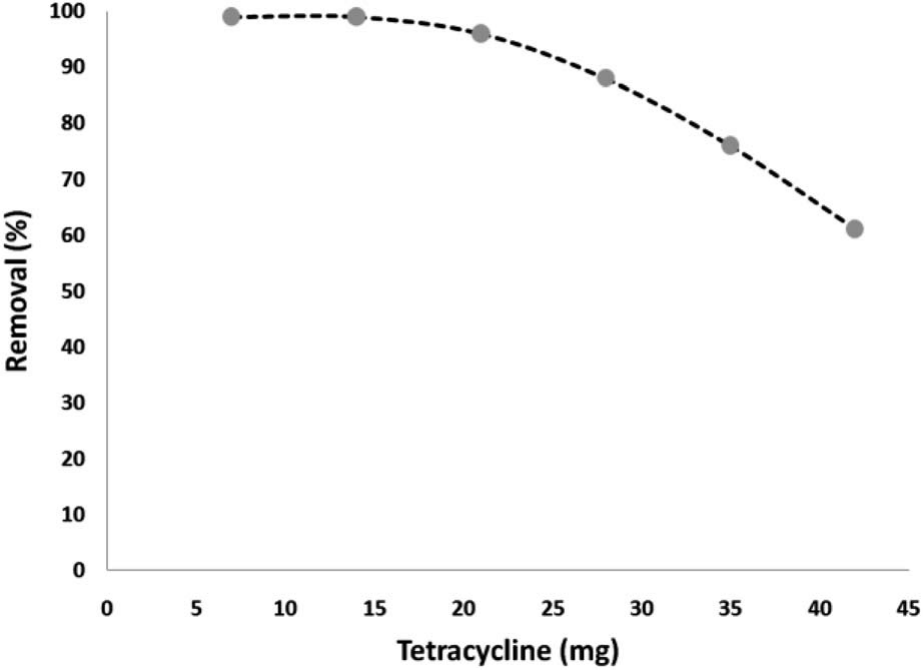
Effect of initial concentration of tetracycline in the presence of UV light.

To verify effect of tetracycline dosage on its degradation percentage, various amounts of tetracycline was used under the optimum conditions. Based on the results, the tetracycline degradation percentage decreased significantly with an increase in its concentration. The tetracycline degradation percentage at 7 and 14 mg L^−1^ at 180 min was at 99%. On the other hand, the degradation percentage at the initial tetracycline concentration of 21, 28, 35, and 42 mg L^−1^ was 96%, 88%, 76%, and 61%, respectively. The increment in photocatalytic degradation with the reduction of tetracycline is a result of the amount of hydroxyl ·OH free radicals present. Under the same pH, CNT-FeNi_3_/DFNS/Cu(ii) MNP dosage and contact time, the amount of hydroxyl ·OH free radicals was equal in the wastewater. According to the results, tetracycline reaction with hydroxyl free radicals increased at a lower dosage, causing the increased removal of tetracycline by free radicals. Also, with the increment in the antibiotic, instead of reaching the surface of all the nanocatalyst particles, the UV light was absorbed by tetracycline, as shown in Fig. 7.

To further investigate the efficiency of the catalyst, different control experiments were performed, and the obtained information is shown in Table 2. Initially, a standard reaction was carried out using FeNi_3_, which showed that the desired product was not formed after 3 h of reaction time (Table 2, entries 1). Also, when FeNi_3_/DFNS and FeNi_3_/DFNS/PEI were used as the catalyst, a reaction was not observed (Table 2, entries 2 and 3). The DFNS or PEI could not give the satisfactory catalytic activity under mild reactions. Based on these disappointing results, we continued the studies to improve the yield of the product by adding Cu(ii) (Table 2, entry 4). The results show that the reaction cycle is mainly catalyzed by Cu(ii) species complexed on the FeNi_3_/DFNS/Cu(ii) nanostructure. The nano-sized particles increase the exposed surface area of the active site of the catalyst, thereby enhancing the contact between the reactants and catalyst dramatically and mimicking homogeneous catalysts. As a result, FeNi_3_/DFNS/Cu(ii) was used in the subsequent investigations because of its high reactivity, high selectivity and easy separation. The results illustrate that amount of catalyst strongly affects the reaction progress and the best result was obtained in the presence of 0.6 mol% of catalyst (Table 2, entry 5). Also, to study the catalytic activity of various FeNi_3_/DFNS/PEI complexes of metal ions as catalysts, we examined their efficiency for the degradation of tetracycline (Table 2). Nine separate reactions were examined in the presence of Cu(ii), Mn(ii), Cd(ii), Co(ii), and Ni(ii) complexes. The results indicated that the catalytic efficiency of Cu(ii) was increased by its immobilization on FeNi_3_/DFNS/PEI.

**Table 2:** Influence of different catalysts for the degradation of tetracycline

Entry	Catalyst	Catalyst loading	Yield^a^ (%)
1	FeNi_3_	5 mg	—
2	FeNi_3_/DFNS	5 mg	—
3	FeNi_3_/DFNS/PEI	5 mg	—
4	FeNi_3_/DFNS/Cu(ii)	5 mg (0.8 mol%)	68
5	FeNi_3_/DFNS/Cu(ii)	5 mg (0.6 mol%)	68
6	FeNi_3_/DFNS/Cu(ii)	5 mg (0.4 mol%)	59
7	FeNi_3_/DFNS/Mn(ii)	5 mg (0.6 mol%)	57
8	FeNi_3_/DFNS/Cd(ii)	5 mg (0.6 mol%)	61
9	FeNi_3_/DFNS/Co(ii)	5 mg (0.6 mol%)	63
10	FeNi_3_/DFNS/Ni(ii)	5 mg (0.6 mol%)	55

a Isolated yield.

**Fig. 8:**
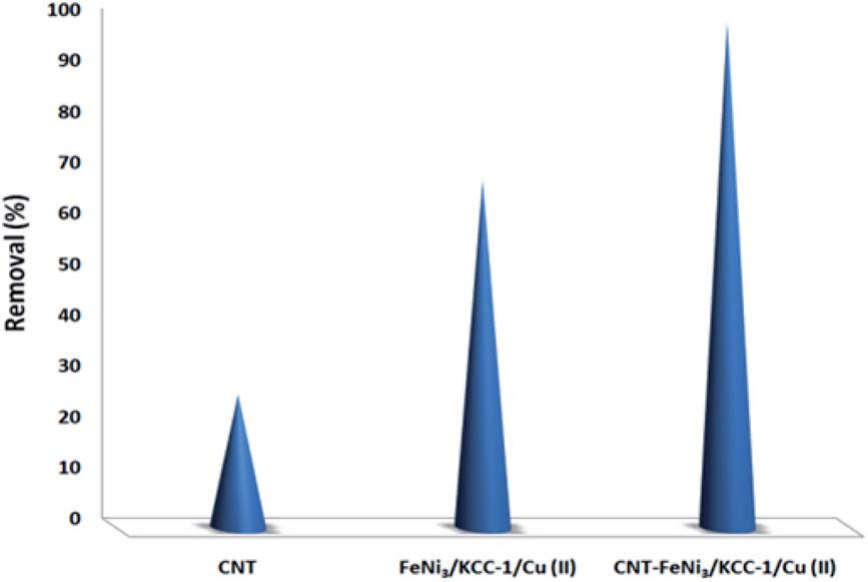
Comparison of the TC removal between adsorption through CNT, photocatalytic degradation with plain FeNi_3_/DFNS/Cu(ii) and photocatalytic degradation with CNT-FeNi_3_/DFNS/Cu(ii) under the same conditions.

**Scheme 3:**
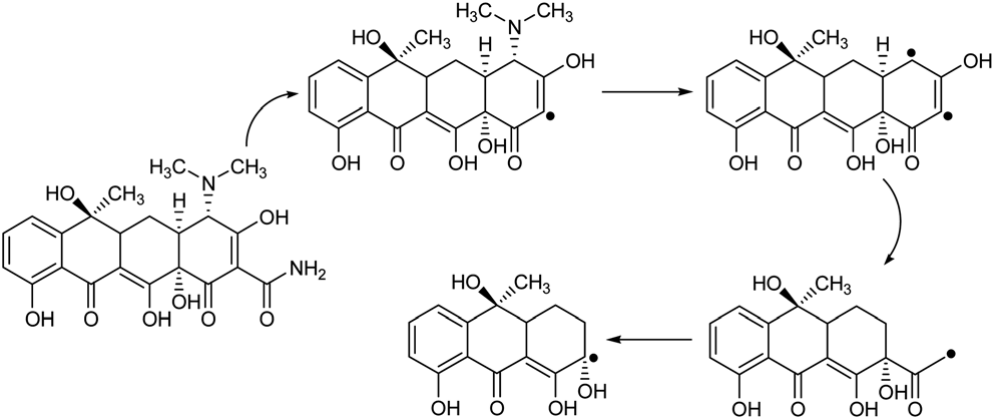
Proposed degradation pathways for the photocatalytic degradation of TC with the CNT-FeNi_3_/DFNS/Cu(ii) photocatalyst.

To determine the influence of adsorption alone, a collection of tests was done in a specific state (initial pH was around 10, dosage of 5 mg L^−1^ CNT-FeNi_3_/DFNS/Cu(ii), irradiation time of 3 h or without UV irradiation, and TC concentration of around 7 mg L^−1^). Fig. 8 shows the obtained results. The removal performance was around 26%, which showed that the effect of adsorption is very low. Thus, photocatalytic degradation is the main process for the removal of TC. Moreover, the effect of plain FeNi_3_/DFNS/Cu(ii) under the optimized conditions without CNT was compared to that of the CNT-FeNi_3_/DFNS/Cu(ii) nanocomposite. Based on its TC degradation performance around 68%, it can be concluded that CNT increased the removal dramatically. The synergistic impact of CNT in comparison with plain FeNi_3_/DFNS/Cu(ii) in TC photodegradation is likely because of the electrons by considering the CNT in the compound, which allow the separation of electron holes.^68^ Moreover, we found that the CNT perform as a photo-sensitizer.^69^ Thus, based on all the experimental results and the previous studies,^70,71^ the possible processes for the degradation are displayed in Fig. S13.† Finally, the intermediate products would be degraded into small inorganic molecular materials (Scheme 3).

The reusability state of a catalyst is known as a significant property in the case of green chemistry. Hence, the reusability of the CNT-FeNi_3_/DFNS/Cu(ii) MNPs was investigated under the optimal conditions for the degradation of TC. After performing the reaction, the solid CNT-FeNi_3_/DFNS/Cu(ii) MNPs were directly separated from the liquid reaction zone magnetically, where in the vicinity of a magnet, the solid readily separates from the solution under a few seconds. After washing with solvent, the catalyst could quickly be reused, as seen in Fig. S13.† Fig. S14† indicates that the catalyst could be reused for 10 consecutive runs. The TC removal was still 95% after the tenth run, which that means only a 4% drop in performance occurred in comparison to that of the fresh catalyst (99%). In addition, the amount of copper leached in the solution for TC degradation after each run was evaluated using ICP. The catalyst exhibited very little leaching during each run, and 0.5% metal leaching was discovered after the tenth run, showing its stability, as illustrated in Fig. S15.†

**Fig. 9:**
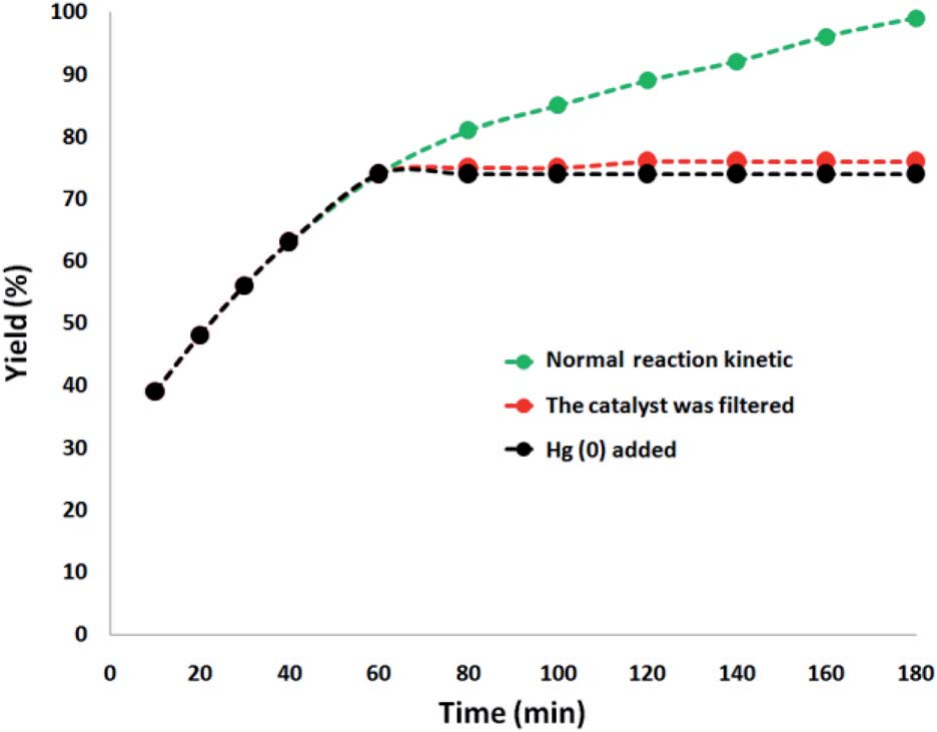
Reaction kinetics, Hg(0) poisoning, and hot filtration studies for the degradation of TC.

In addition, a complete study was performed to clarify the heterogeneous nature of the catalyst. Firstly, we performed a hot filtration experiment for the degradation of TC under the optimum conditions and determined that the catalyst was magnetically removed *in situ* after around 74% removal of TC occurred after 1 h. In addition, the reactants were allowed to undergo more reactions. The results demonstrated that after removing the heterogeneous catalyst, the free catalyst remnant was feeble active, and the conversion of about 76% was obtained after 3 h of TC degradation. This proved that the catalyst worked heterogeneously during the reaction and only a slight amount of leaching occurred during the reaction. Secondly, to ensure the heterogeneous nature of the catalyst, a mercury poisoning experiment was additionally performed. Mercury(0) is absorbed on a metal and dramatically deactivates the active surface of a metal catalyst, thus reducing its catalytic activity. The conducted experiment proved the composite is a heterogeneous catalyst. This test was performed with the model of reaction the under optimal conditions. After 1 h of reaction, about 300 molar mercury was released to the reaction compound. The reaction mixture was stirred for more than 3 h. In this reaction, no further conversion was seen after 120 min due to the catalyst being poisoned. A kinetic scheme of the reaction in the presence of Hg(0) is demonstrated in Fig. 9. The negative outcomes obtained from the heterogeneity experiments (Hg(0) poisoning and hot filtration) indicated that the solid catalyst is really heterogeneous and no acquirable copper leaching occurred during the degradation of TC.

**Fig. 10:**
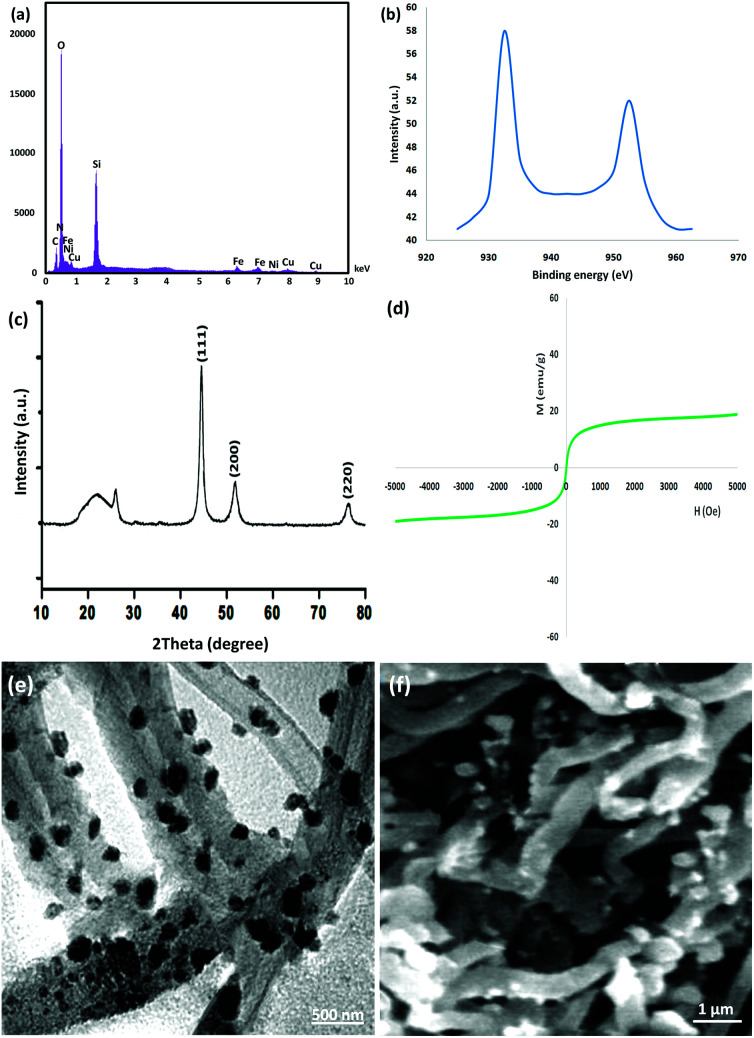
(a) EDX and (b) XPS spectra, (c) XRD, (d) VSM, (e) TEM, and (f) FE-SEM images of the recovered CNT-FeNi_3_/DFNS/Cu(ii) MNPs after the 10th run for the photocatalytic degradation of tetracycline.

Eventually, to ensure that the structure of the recovered catalyst was maintained, it was characterized after the 10th of photocatalytic degradation of tetracycline in wastewater under the determined optimum conditions, as seen in Fig. 10. The EDX pattern of the recycled catalyst demonstrated the presence of all its components, verifying its stability during the reactions, as observed in Fig. 10a. The XPS pattern demonstrated that the Cu element in the recycled catalyst after the 10th run was same as that of the fresh catalyst. As seen in Fig. 10b, no other oxidation states were detected in the catalyst. As seen in Fig. 10c, the XRD pattern of the reproduced catalyst proved that the structure of the used catalyst was totally intact after recycling. The recovered catalyst exhibited an outer magnetic zone and can be easily separated from the reaction mixture just as the fresh catalyst (refer to Fig. 10d). The TEM picture indicated that the generic white and gray dots on the straight chain of CNT were FeNi_3_/DFNS/Cu(ii) after the 10th run (refer to Fig. 10e). Notably, the nanocatalyst did not exhibit any morphological variations, as proven by the FE-SEM images of the recovered catalyst (refer to Fig. 10f).

## Conclusions

We successfully synthesized CNT-FeNi_3_/DFNS/Cu MNPs. Herein, the catalyst was characterized in detail *via* XPS, XRD, SEM, FTIR, CV, TGA, TEM, EDX, UV-Vis, VSM, ICP, and BET. We expanded a novel stable, low-cost, and non-toxic catalytic system for removing tetracycline by producing CNT-FeNi_3_/DFNS/Cu MNPs. The CNT-FeNi_3_/DFNS/Cu MNPs exhibited a good performance for the degradation of tetracycline in an aqueous environment. The CNT-FeNi_3_/DFNS/Cu nanocomposite exhibited a superparamagnetic nature, which particularly, simplified the separation of the catalyst from the reaction mixture utilizing a magnet. In this regards, the catalyst remained stable under the reaction conditions and retained its catalytic activity and selectivity for ten successive runs without the requirement of its re-activation, which alleviates economic and environmental problems. In addition, hot filtration together with mercury poisoning tests confirmed the nature of the catalyst and its negligible metal leaching.

## Conflicts of interest

There are no conflicts to declare.

## Supplementary Material

RA-009-C9RA05817F-s001
