# Supplementary material for: Synthesis and characterization of a novel CNT-FeNi3/DFNS/Cu(ii) magnetic nanocomposite for the photocatalytic degradation of tetracycline in wastewater
Source: RSC Adv. 2019 Oct 30;9(60):35022–32. doi: 10.1039/c9ra05817f (PMC9074154; doi:10.1039/c9ra05817f)
Supplement: RA-009-C9RA05817F-s001 [file RA-009-C9RA05817F-s001.pdf]

Electronic Supplementary Material (ESI) for RSC Advances.
This journal is © The Royal Society of Chemistry 2019

Synthesis and characterizations of a novel CNT-FeNi$_3$/DFNS/Cu (II) magnetic nanocomposite for photocatalytic degradation of tetracycline in wastewater

Yanhua Zhao[a], Jie Juan Tang*[b], Alireza Motavalizadehkakhky*[c], Saeid Kakooei[d], Seyed Mohsen Sadeghzadeh[e,f]

[a]*School of Economics and ManagementLangfangNormal University, Langfang,065000, China.*
[b]*School of Humanities, Tianjin Agricultural University, Tianjin 300384,China.*
[c]*Department of Chemistry, Faculty of Sciences, Neyshabur Branch, Islamic Azad University, Neyshabur, Iran.*
[d]*Centre for Corrosion Research, Department of Mechanical Engineering, Faculty of Engineering, Universiti Teknologi PETRONAS, 32610 Seri Iskandar, Perak Darul Ridzuan, Malaysia.*
[e]*New Materials Technology and Processing Research Center, Department of Chemistry, Neyshabur Branch, Islamic Azad University, Neyshabur, Iran.*
[f]*Young Researchers and Elite Club, , Neyshabur Branch, Islamic Azad university, Neyshabur , Iran.*

E-mail: amotavalizadeh@yahoo.com (A. Motavalizadehkakhky); daishuxue2000@126.com (J.J. Tang).

Supporting Information

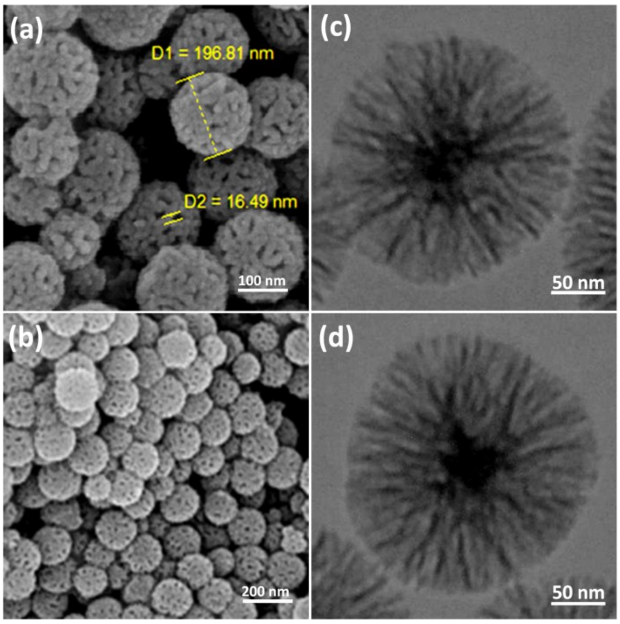

Figure S1 TEM images of FeNi$_3$/DFNS MNPs (a); FeNi$_3$/DFNS/Cu (II) complexes MNPs (b); FE-SEM images of FeNi$_3$/DFNS MNPs (c); FeNi$_3$/DFNS/Cu (II) complexes MNPs (d).

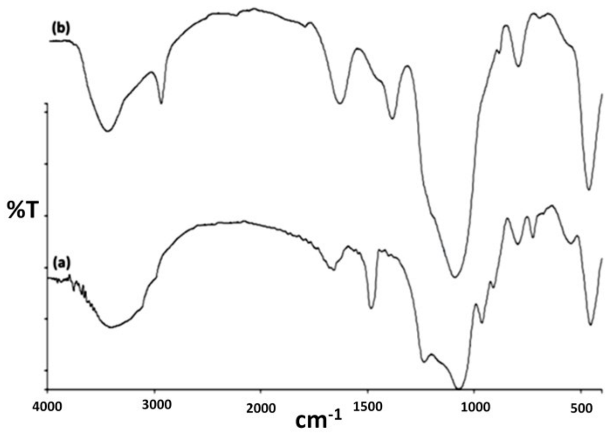

Figure S2 FTIR spectra of (a) $FeNi_3$/DFNS MNPs, and (b) $FeNi_3$/DFNS/PEI MNPs.

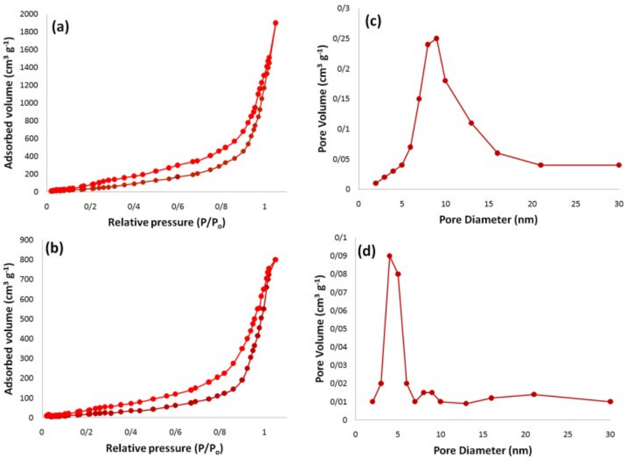

Figure S3 adsorption-desorption isotherms of the $FeNi_3$/DFNS NPs (a); $FeNi_3$/DFNS/Cu (II) complexes MNPs (b); and BJH pore size distributions of the $FeNi_3$/DFNS NPs (c); $FeNi_3$/DFNS/Cu (II) complexes MNPs (d).

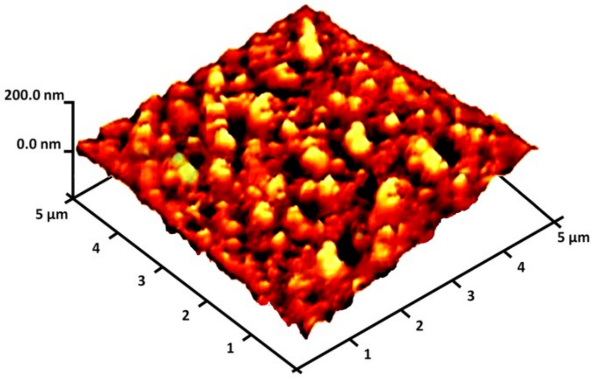

Figure S4 Three-dimensional of AFM images of $FeNi_3$/DFNS/Cu (II) complexes MNPs.

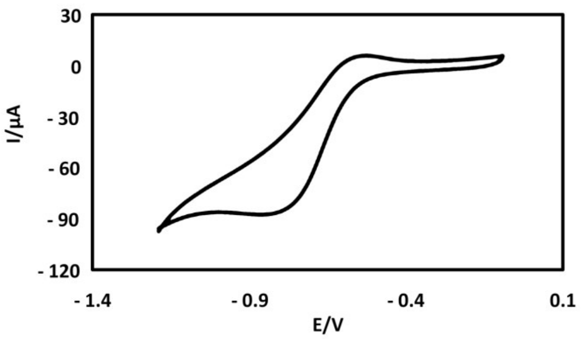

Figure S5 Cyclic voltammogram of copper(II) complex.

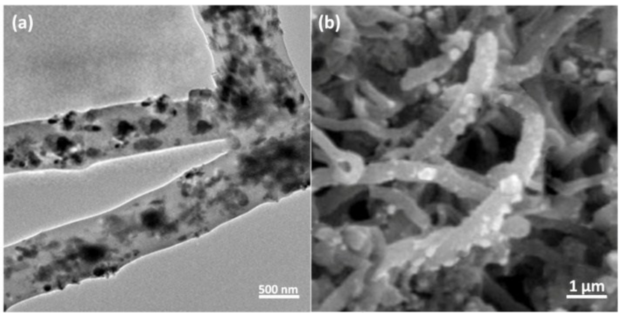

Figure S6 SEM images of a) CNT-$FeNi_3$/DFNS/Cu MNPs, and TEM images of b) CNT-$FeNi_3$/DFNS/Cu MNPs.

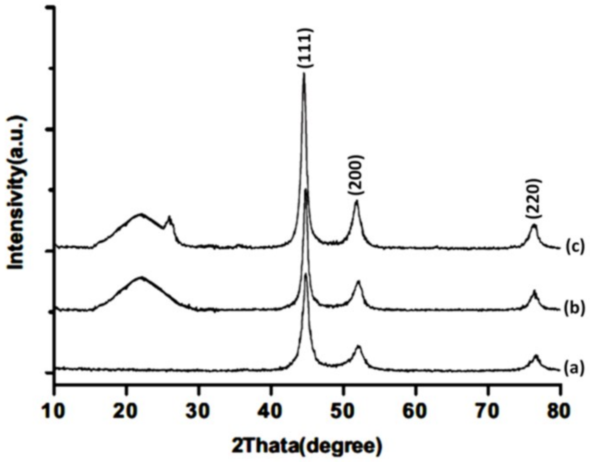

Figure S7 XRD analysis of (a) $FeNi_3$, (b) $FeNi_3$/DFNS/Cu (II), and (c) CNT-$FeNi_3$/DFNS/Cu (II) complexes MNPs.

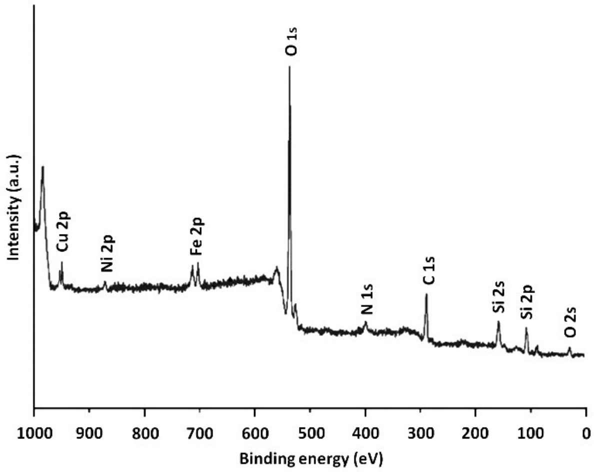

Figure S8 XPS spectra of CNT-$FeNi_3$/DFNS/Cu MNPs.

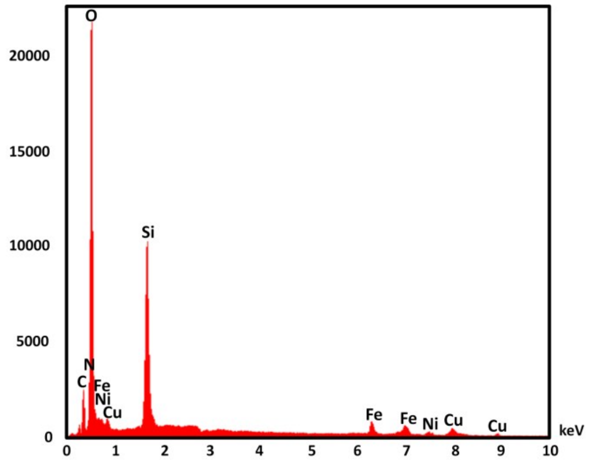

Figure S9 The EDX spectra of CNT-$FeNi_3$/DFNS/Cu MNPs.

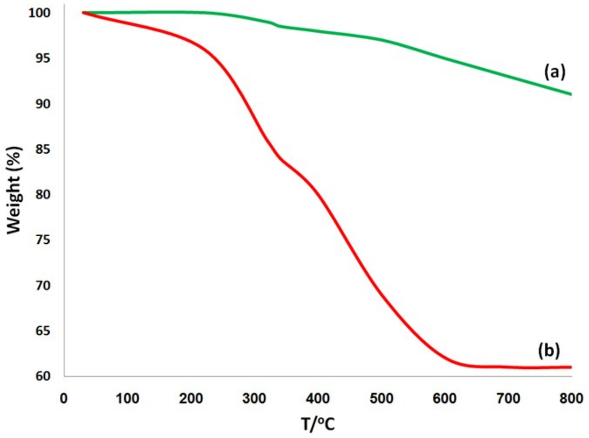

Figure S10 TGA curve of (a) raw CNTs, and (b) CNT-$FeNi_3$/DFNS/Cu MNPs.

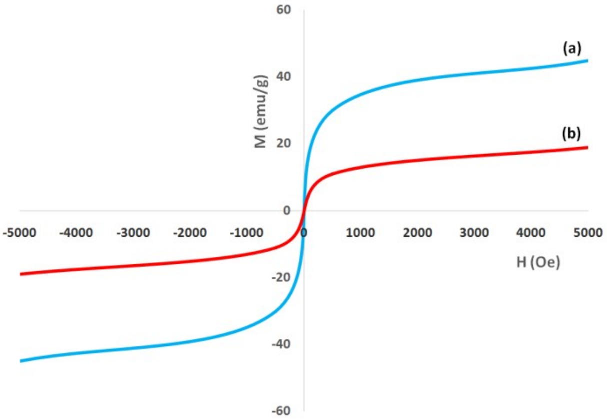

Figure S11 Room-temperature magnetization curves of (a) $FeNi_3$/DFNS/Cu (II), and (b) CNT-$FeNi_3$/DFNS/Cu (II) complexes MNPs.

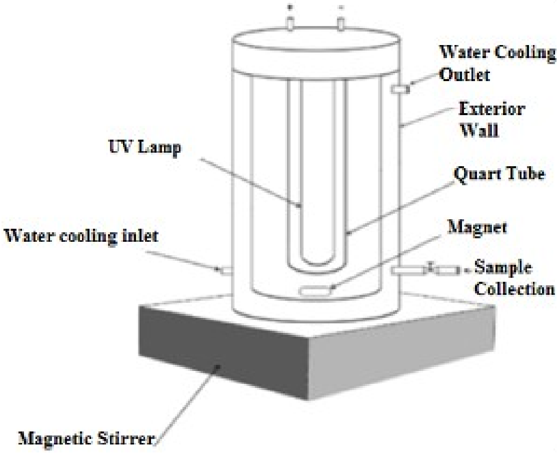

Figure S12 Schematic of the photocatlytic reactor.

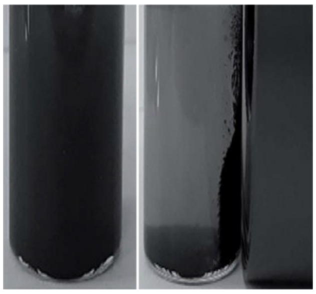

Figure S13 Catalytic separations by magnetic attraction.

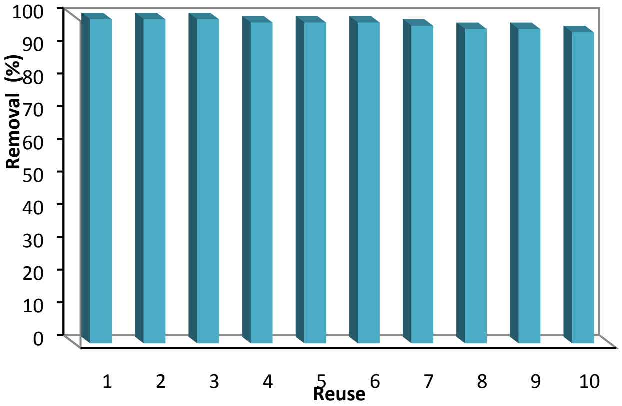

Figure S14 The reusability of catalysts for degradation of TC.

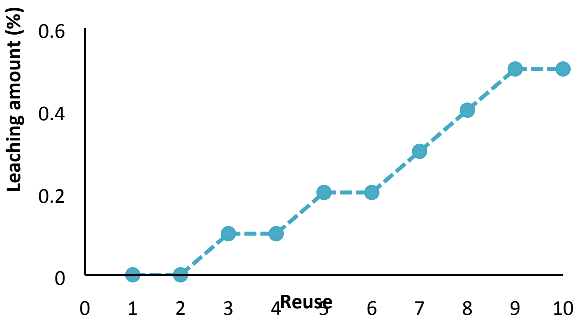

Figure S15 Recyclability of the catalyst for degradation of TC.
